# The Muscle-Brain Axis and Neurodegenerative Diseases: The Key Role of Mitochondria in Exercise-Induced Neuroprotection

**DOI:** 10.3390/ijms22126479

**Published:** 2021-06-17

**Authors:** Johannes Burtscher, Grégoire P. Millet, Nicolas Place, Bengt Kayser, Nadège Zanou

**Affiliations:** 1 Institute of Sport Sciences, University of Lausanne, CH-1015 Lausanne, Switzerland; gregoire.millet@unil.ch (G.P.M.); nicolas.place@unil.ch (N.P.); bengt.kayser@unil.ch (B.K.); nadege.zanou@unil.ch (N.Z.); 2 Department of Biomedical Sciences, University of Lausanne, CH-1005 Lausanne, Switzerland

**Keywords:** muscle, exercise, brain, mitochondria, myokines, neurodegeneration

## Abstract

Regular exercise is associated with pronounced health benefits. The molecular processes involved in physiological adaptations to exercise are best understood in skeletal muscle. Enhanced mitochondrial functions in muscle are central to exercise-induced adaptations. However, regular exercise also benefits the brain and is a major protective factor against neurodegenerative diseases, such as the most common age-related form of dementia, Alzheimer’s disease, or the most common neurodegenerative motor disorder, Parkinson’s disease. While there is evidence that exercise induces signalling from skeletal muscle to the brain, the mechanistic understanding of the crosstalk along the muscle–brain axis is incompletely understood. Mitochondria in both organs, however, seem to be central players. Here, we provide an overview on the central role of mitochondria in exercise-induced communication routes from muscle to the brain. These routes include circulating factors, such as myokines, the release of which often depends on mitochondria, and possibly direct mitochondrial transfer. On this basis, we examine the reported effects of different modes of exercise on mitochondrial features and highlight their expected benefits with regard to neurodegeneration prevention or mitigation. In addition, knowledge gaps in our current understanding related to the muscle–brain axis in neurodegenerative diseases are outlined.

## 1. Introduction

The umbrella term “neurodegenerative disease” refers to the progressive pathological loss of neurons in specific neuronal circuits. Examples are Huntington’s disease, amyotrophic lateral sclerosis, spinocerebellar ataxia, and Alzheimer’s (AD) and Parkinson’s diseases (PD). AD and PD are the most common causes of dementia and of neurodegenerative motor diseases, respectively. Their global prevalence has been estimated to be about 30–40 million people suffering from AD in 2019 [1] and about 6.1 million people (2016) [2] from PD. These prevalences are predicted to increase massively in the next decades [3]. The vast majority of both PD and AD cases are sporadic, with age being the most important risk factor for disease development [4].

Currently no treatment strategies that modify the disease course of AD or PD exist, and symptomatic treatments are limited, in particular for AD. Lifestyle factors, notably including physical activity and exercise, however, are major factors in modulating the risk of developing these neurodegenerative diseases [5,6,7]. Exercise is defined as planned, structured, and repetitive physical activity that is performed with the objective to improve or maintain physical fitness [8]. An important aspect of both general physical activity and exercise is the activation and training of skeletal muscles, which is associated with enhanced mitochondrial function. Regular endurance exercise improves cardiorespiratory fitness and skeletal muscle function and has well-documented health effects, including reduced all-cause mortality [9]. It furthermore importantly influences mitochondrial health [10,11].

Here, we review the evidence for signalling between skeletal muscle and the brain contributing to the neuroprotective effect of exercise. We focus on the muscle–brain crosstalk mediated by mitochondria for the beneficial effect of exercise in AD and PD. The convergence of pathogenic mechanisms in many neurodegenerative diseases that include mitochondrial dysfunction and oxidative stress [12,13], protein aggregation [14,15], inflammation [16], and brain regional vulnerabilities [17] also highlight the relevance of the related influences of muscle–brain crosstalk for other neurodegenerative diseases.

## 2. Mitochondrial Dysfunction in Neurodegenerative Diseases

Despite the highly divergent pathology and symptomatology associated with different neurodegenerative diseases, several intriguing commonalities exist. For example, mitochondrial dysfunction (which may include deficits in mitochondrial metabolism, respiration, dynamics, redox regulation, ion homeostasis, and cell death regulation) is at the core of the aetiology of most neurodegenerative diseases [12,13]. Mitochondrial abnormalities have, e.g., been described early in the substantia nigra of PD [18] and in the cortex of AD [19] and Huntington’s disease [20] patients.

Even if it is unclear whether mitochondrial dysfunction is a cause or consequence of neurodegenerative diseases, it certainly is a determining factor of disease progression. Based on their multitude of functions in the cell, mitochondria affect the cellular fate in different ways. Some of the main functions of mitochondria that are thought to be compromised in neurodegeneration are summarized in the following sections.

### 2.1. Mitochondrial Respiration and Energy Provision

The brain has high energy requirements for which it mostly relies on oxidative energy metabolism [21]. It therefore critically depends on continuous adequate oxygen and substrate supplies. Mitochondrial oxidative phosphorylation (OXPHOS) is the most important energy-providing process in the brain. OXPHOS consists of the electron transport system (mitochondrial complexes I–IV) that establishes a proton gradient across the inner mitochondrial membrane. The resulting mitochondrial membrane potential is used by the second OXPHOS component, the phosphorylation system, to generate and transport ATP. Dysfunction of distinct complexes of the respiratory chain traditionally have been linked to different neurodegenerative diseases. Thus, for example complex I dysfunction has been attributed a prominent role in PD pathogenesis [12], complex IV in AD [12], and complex II in Huntington’s disease [22,23]. It is, however, becoming increasingly clear that significant individual differences in mitochondrial dysfunctions exist even within specific neurodegenerative disease categories and that numerous mitochondrial components can be defective in all of these categories, as recently shown for PD [24].

### 2.2. Mitochondrial ROS and Oxidative Stress

Oxygen, even though essential for human life, can in some conditions be highly reactive and therefore a potentially dangerous element. The formation of reactive oxygen species (ROS) is part of aerobic metabolism, and a delicate balance between too little and too much defines cellular homeostasis [25]. Oxidative stress represents an imbalance between oxidants and antioxidants in favour of the oxidants. Moderate elevations in ROS levels are considered physiological, since they are implicated in protective redox signalling and redox regulation [25,26] and are also required for beneficial adaptations to exercise [27,28]. ROS production during oxidative phosphorylation can be aggravated by mitochondrial damage [29]. In contrast, supraphysiological high mitochondrial ROS levels result in the oxidative damage of multiple biomolecules, such as DNA, lipids, and proteins. This impairs cellular processes, such as the maintenance of membrane potential, trans-membrane transport, proteostasis, and enzyme activities, and is also implicated in the pathogenesis of many diseases [30,31]. Oxidative stress is a core mechanism in neurodegenerative disease pathogenesis [13]. This is reflected, for example, by increased levels of oxidative modifications in the substantia nigra of PD patients [32] and oxidative stress as an early pathological process in AD [33].

### 2.3. Mitochondrial Biogenesis

The increase of mitochondrial mass from pre-existing mitochondria (mitochondrial biogenesis) is controlled by numerous pathways and revolves around the cotranscriptional factor peroxisome-proliferator-activated receptor γ coactivator-1α (PGC-1α) and other molecules [27,34]. The PGC-1α-mediated activation of mitochondrial transcription factor A induces mitochondrial growth and division to generate new mitochondria [35]. An important regulator of mitochondrial biogenesis is the exercise-inducible PGC-1α activator AMP-activated protein kinase (AMPK) [35]. The reduced responsiveness of AMPK signalling in aging [36] may be a determinant of increased vulnerability to neurodegenerative disease, not only due to its effects on mitochondrial biogenesis but also on its modulatory role in inflammatory responses, antioxidative defences, and autophagy [36,37]. Impairments of mitochondrial biogenesis are implicated in the pathogenesis of several neurodegenerative diseases and are increasingly considered for treatment strategies in such disorders [38]. Deficits of mitochondrial biogenesis have been shown for both AD [39] and PD [40].

### 2.4. Mitochondrial Dynamics and Mitophagy

Mitochondria are highly dynamic organelles, which can change their number and size by fusion and fission [41]. Fusion denotes the process of several smaller separated mitochondria merging into one bigger mitochondrion, while fission refers to the division of one mitochondrion into several smaller mitochondria. In contrast to mitochondrial biogenesis and mitophagy, these processes are not associated with a change in mitochondrial mass but also influence mitochondrial function. Fusion of the outer mitochondrial membrane is effectuated primarily by mitofusin 1 (Mfn1) and Mfn2 and fusion of the inner mitochondrial membrane by optic atrophy 1 (Opa1). Mitochondrial fission is executed by dynamin-related protein 1 (Drp1), a cytosolic protein that is recruited to the mitochondrial surface in response to various physiological cues [42]. The morphological changes have functional implications; enhanced fusion facilitates oxidative phosphorylation and the intermitochondrial exchange of metabolites and mitochondrial DNA [43]. Fission enables more efficient mitophagy [44]. Mitophagy ensures mitochondrial quality via the clearance of damaged (e.g., impaired mitochondrial membrane potential or oxidative phosphorylation and mitochondrial DNA mutations [45]) mitochondria by autophagy and lysosomal degradation. Additionally, mitochondrial trafficking within the cell—for example, for cell membrane repair [46]—depends on fission. Alterations of mitochondrial dynamics are suspected to be involved in the neurodegenerative process and have been demonstrated in models for all major neurodegenerative diseases, including AD [47] and PD [48]. Missense mutations in proteins involved in mitochondrial dynamics, furthermore, are associated with parkinsonism and dementias, such as AD [49].

The efficient clearance of dysfunctional mitochondria is crucial for cell survival. Damaged mitochondria pose a threat to cells as they release excess ROS and inflammatory and cell-death related factors, and this threat may be increased in neurodegenerative diseases: impaired mitophagy has, for example, been demonstrated in models of AD [50]. Familial forms of PD can be caused by mutations in the mitophagy-related genes PTEN-induced kinase 1 (PINK1) [51] and Parkin [52], which is a strong indication for the role of defective mitophagy in PD.

### 2.5. Mitochondrial Control of Cell Death and Survival

Mitochondria are integrally involved in the control of cellular survival. One important mechanism mediating this control is the mitochondrial membrane potential that regulates mitochondrial import and export and, upon deterioration, can trigger mitochondrial cell death signalling [53] and induce mitophagy, apoptosis, or necrosis. While the clearance of damaged or senescent cells is important to maintain a healthy cellular environment and to limit mitochondrial damage signalling, too much cell death is obviously detrimental. Neurons are in general vulnerable to imbalances in cell death signalling based on their postmitotic nature that renders them difficult to replace; this vulnerability increases with age, and certain neuronal populations are more vulnerable than others [17], which is likely an important factor in the pathogenesis of neurodegenerative diseases.

In summary, functional mitochondria are important to maintain brain health. The exacerbation of mitochondrial DNA mutations and oxidative stress, as well as of deficits in mitochondrial biogenesis, dynamics, and quality control [35,54,55,56,57,58] during aging likely contributes to the increased risk of neurodegenerative disease at an older age. The capacity of exercise to improve mitochondrial function will be discussed in the next sections.

## 3. Improving Mitochondrial Functions by Exercise in Skeletal Muscle

The prominent health effects of regular exercise are well established and include reduced all-cause morbidity and mortality [9]. Regular exercise slows down [59,60] age-related decreases [61] of cardiorespiratory fitness. The general antiaging effects of exercise have recently been reviewed by Radak et al. [62].

With regard to mitochondria, exercise benefits are best understood in skeletal muscle. While muscle mitochondria regulate skeletal muscle mass and function [63], they are in turn regulated by exercise. Exercise induces mitochondrial plasticity [27,64,65] and improves mitochondrial biogenesis and respiration. It also enhances antioxidant capacities and the affinity of mitochondria for oxygen [27,66,67,68,69,70], improving fatty acid oxidation, aerobic performance, health [71,72,73,74], and healthy aging [75]. These mechanisms crucially depend on the mitochondrial integrity and quality control, as well as on the mitochondria’s capacity to adequately change their morphology, increase their numbers, and enhance their mobility and distribution throughout cells.

Hormetic adaptations are thought to be important mediators of exercise-induced mitochondrial benefits [76]. “Hormesis” is a biphasic response to a stimulus (here exercise), which results in protective adaptations at low levels but that can be detrimental at high doses. It is termed “mitohormesis” if mitochondrial adaptations are concerned. Hormesis following exercise has been suggested to promote healthy brain aging [77]. According to the nature of hormetic effects, however, doses of exercise that are too high have been shown to be detrimental for mitochondria [78], further discussed in an associated editorial [79].

Muscle mitochondrial ATP production has been shown to decrease by around 8% per 10 years of higher age in a population of 146 healthy men and women of ages between 18 and 89 years [80]. This deterioration of mitochondrial function was attributed mainly to physical inactivity [81], suggesting that regular exercise may at least partially prevent the aging-related decline of mitochondrial function. While acute exercise induces the formation of ROS, with oxidative damage occurring more frequently during high intensity bouts [82], regular moderate exercise reduces oxidative stress by bolstering the antioxidant defences of muscle tissue [30,83,84,85]. Accordingly, aging-related deficiencies in antioxidant capacities can be mitigated by life-long regular exercise [83]. One of the most thoroughly investigated effects of mitochondrial adaptations in response to exercise is mitochondrial biogenesis. A single bout of high intensity exercise is enough to upregulate proteins involved in mitochondrial biogenesis and energy production [69], to induce the translation of oxidative phosphorylation-linked proteins [69], and to improve ATP generation [27] in skeletal muscle. In combination with increased mitochondrial protein synthesis and the induction of mitochondrial fusion, this leads to increased mitochondrial biogenesis and efficiency [69]. Higher exercise volumes [70], multiple exercise sessions per day, and elevated intensity [69] are associated with increasing stimulation of mitochondrial protein synthesis and mitochondrial fusion, improving structural and functional mitochondrial features. Mechanistically, exercise-induced AMPK [35] and PGC-1α [86] upregulation are thought to mediate mitochondrial biogenesis effects.

PGC-1α furthermore contributes to the anti-inflammatory effects of physical activity [87]. Exercise can promote mitochondrial fusion and inhibit aggravated fission [88]. Mitochondrial fusion is thought to increase respiratory efficiency [43], while pronounced mitochondrial fission is linked to aging and to age-related disease [89].

Acute moderate intensity exercise induces mitochondrial fusion in muscle tissue and high intensity bouts increase it even further [69,90]. Chronic exercise also leads to an enhanced expression of the pro-fusion proteins Opa1 and Mfn2 and to a reduced expression of the pro-fission protein Drp1 [27]. Mice performing lifelong voluntary exercise showed reduced age-related mitochondrial fragmentation [91].

Stimulation of mitophagy by acute and chronic exercise has been demonstrated in animal experiments [27], and while this effect is also thought to occur in humans, it may be most robust as a result of lifelong, as opposed to transient chronic, exercise [92].

**Figure 1:**
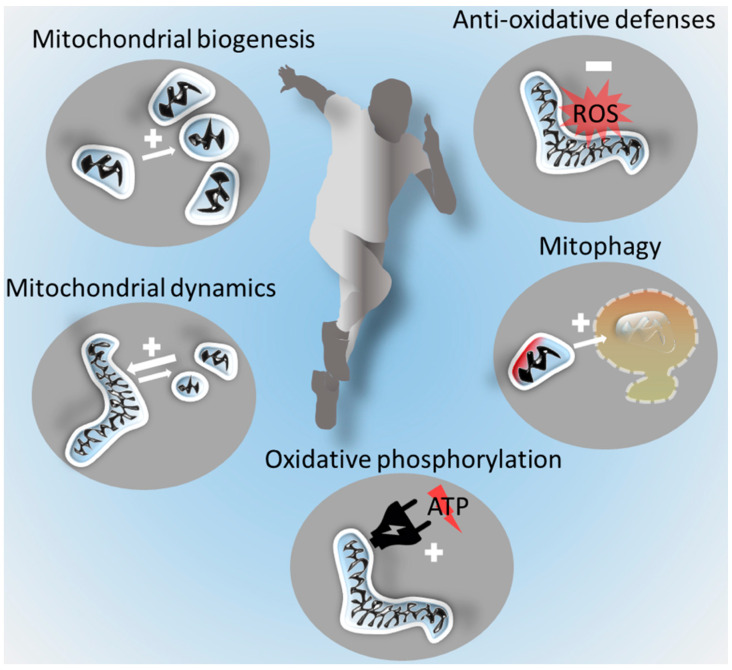
Exercise-induced benefits on skeletal muscle mitochondria. Regular moderate exercise promotes the indicated mitochondrial functions. ROS: reactive oxygen species; ATP: adenosine triphosphate.

In summary, regular exercise has the capacity to improve both mitochondrial structure and function in skeletal muscle: the main effects are summarized in Figure 1.

## 4. Exercise Effects on the Brain: A Focus on Mitochondria and Oxidative Stress

The brain is well known to be responsive to regular exercise. Pronounced structural changes have recently been confirmed by the demonstration of differential brain volumes depending on exercise levels [93]. The associated aerobic fitness is further correlated with reduced brain tissue loss [94,95] and with cognitive benefits [96] during the aging process. Accordingly, exercise has emerged as an important protective factor for neurodegenerative diseases, such as AD [5] and PD [6,97].

The vulnerability of the brain to energy deficits, oxidative stress, and dysregulated cell-death signalling becomes even more pronounced with advancing age, when the occurrence of mitochondrial dysfunction, oxidative damage, and impaired molecular waste disposal increases [98]. Unsurprisingly, age is thus a central risk factor for the development of sporadic neurodegenerative diseases [4]. While potentially attenuated at a higher age [27], the beneficial effects of mitochondrial adaptations to exercise occur throughout life [99].

An obvious benefit of physical activity and exercise with respect to neurodegenerative diseases is the reduction of cardiovascular risk factors [100]. However, the brain can also benefit from exercise and improved muscle function in ways that suggest a direct muscle–brain crosstalk [101,102], and we hypothesize that mitochondria are central in this crosstalk.

Mitochondrial plasticity in response to exercise is best known in skeletal muscle, but it has also been reported for other tissues, including the brain [103,104,105,106,107]. Similar to the effects in skeletal muscle, the exercise-induced enhancement of the mitochondrial electron transport system [108], biogenesis [103], and antioxidative capacities [108] has been reported in the mouse brain. Exercise-induced transient increases in ROS levels can affect the redox regulation, even in the brain [109,110]—as in skeletal muscle—and also induce adaptive responses therein, enhancing endogenous antioxidant capacities [111]. These adaptations, already observed following single bouts of exercise, are reinforced by regular exercise by increasing cellular antioxidative and repair capacities, inducing an increased tolerance to oxidative stress [112]. For example, chronic (daily, for 15 weeks) moderate treadmill exercise reduced ROS levels and protein carbonyls and increased superoxide dismutase 1 and glutathione peroxidase in the hippocampus of adult female rats [104]. Regular exercise likely protects from neurodegenerative disease both by an upregulation of antioxidative stress defences and related beneficial mitochondrial adaptations as well as indirectly by the regulation of neuroprotective factors, such as brain-derived neurotrophic factor (BDNF) via ROS [111].

As much as is understood about brain–muscle communication through efferent and afferent neuronal signalling, little is yet understood about humoral exercise signalling between the muscle and the brain. In the next section, we will summarize some important routes of the muscle–brain axis that are likely involved in such communication with a focus on the potential role of mitochondria.

## 5. How Do Muscles Communicate with the Brain?

Exercise induces muscle adaptations that affect remote tissues. The mechanistic underpinnings of this communication are still not fully understood (reviewed by [113] and [114]) but are thought to mainly involve endocrine signalling of skeletal muscle [105] by exercise-induced myokines [102,115]. Many of these molecules travel systemically (in blood or lymph) via extracellular vesicles that are today considered as key messengers of paracrine exercise signals [116,117,118,119], as they have been described to be released from skeletal muscle only in 2015 [120]. Apart from myokines, they transport various other bioactive molecules, such as proteins and microRNAs [116,118].

### 5.1. Exercise-Induced Alteration of Systemic Parameters

Exercise probably also contributes to the metabolic and mitochondrial “reprogramming” of remote tissues via its effect on systemic parameters, such as temperature, hypoxia, blood pressure, or pH, which are sensed, e.g., by baro- and chemoreceptors [121]. The regulation of the cerebral blood flow (CBF) is likely involved in such reprogramming. CBF increases during exercise [122,123], which is necessary to maintain brain oxygenation. The brain is much more vulnerable to drops in oxygenation than skeletal muscle [116], and an inappropriate oxygen supply would compromise brain function and increase risk of long-term damage. A tight control of CBF is important due to the highly regulated permeability across the blood–brain barrier (BBB), changing oxygen demands with neuronal activation and the particular vulnerability of the brain’s microvasculature [124]. The enhanced general clearance mechanisms of harmful substances from the brain in response to exercise might also contribute to a reduction of damaged mitochondria. This may result from enhanced CBF [122,123], following increased activity of the glymphatic system during sleep due to exercise [125]. The glymphatic system allows clearance of waste products via the cerebrospinal fluid through the interstitial space into the peripheral circulation [126].

While it is debated whether CBF declines with age, chronically reduced CBF in clinical cohorts has been linked to cognitive decline, and it has been speculated that exercise protects from this vulnerability [127].

The potentially neuroprotective outcome of these exercise-induced signals to the brain, in turn, contributes to increased or maintained brain function, including improved cognitive function associated with the hippocampus [128] and improved resilience of hypothalamic neurons involved in hormonal control of hunger and satiety to detrimental high-fat diet effects [106]. The mitochondrial reprogramming, improved waste clearance, and enhanced nutrient and oxygen supply in response to exercise may increase mitochondrial functioning as a potential mechanism of exercise benefits for the brain.

### 5.2. Myokines

Exercise exerts pronounced effects directly on skeletal muscle tissues but also on distal tissues, including the cardiovascular, pulmonary, metabolic, and neuroendocrine systems. These adaptations are initiated from muscle tissue through different paracrine factors such as nitric oxide (NO), ATP, ROS [129], or myokines, defined as “…cytokines or other peptides that are produced, expressed and released by muscle fibres” [102]. The term “exerkines” has been proposed to more broadly define peptides and nucleic acids that are released in response to exercise from skeletal muscle and other organs [130].

**Figure 2:**
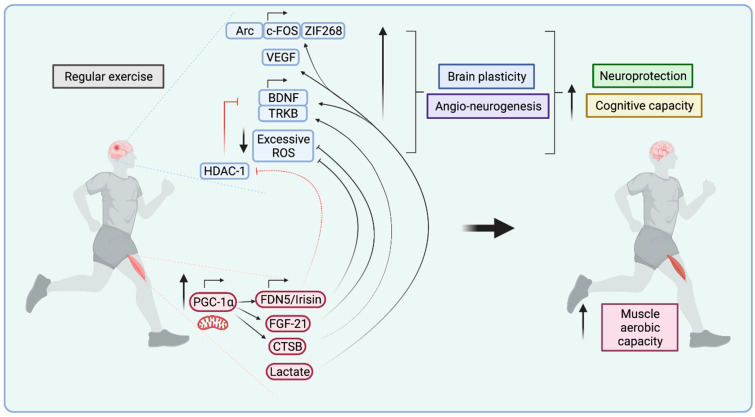
Exercise-induced neuroprotection through circulating myokines. Exercise induces increased peroxisome-proliferator-activated receptor γ coactivator-1α (PGC-1α) expression and supports induction of different myokines such as FDN5/Irisin, cathepsin B (CTSB), fibroblast growth factor 21 (FGF21), and lactate. FDN5/Irisin induces brain-derived neurotrophic factor (BDNF) expression in the brain through indirect inhibition of histone deacetylase 1(HDAC-1). Several of these factors increase signalling of BDNF and its receptor Tropomyosin receptor kinase B (TRKB), or reduce excessive reactive oxygen species (ROS) production. In addition, lactate increases the expression of the vascular endothelial growth factor (VEGF) and of the synaptic plasticity genes: Arc, c-FOS and ZIF268.

The release or uptake of these substances from and into contracting muscle cells allows tissue interactions, including crosstalk between skeletal muscle and the brain. Contracting muscle fibres produce and release myokines [131] that play an important role in skeletal muscle crosstalk with other organs and tissues [132]. The functional consequences of their release are determined by factors such as exercise volume, intensity, and frequency [113]. Numerous exercise-induced myokines have been identified, among them irisin, cathepsin B, fibroblast growth factor 21 (FGF-21), BDNF, and many more (for review, see [133]), and together, these molecules have been termed the “myokinome” [132]. They all participate in systemic exercise signalling, and some of them have been specifically linked to central nervous system effects by modulating, for example, adult neurogenesis and cognitive function. This suggests that these myokines at least partially mediate brain benefits in response to regular exercise. Although such benefits include well-defined outcomes, for example, on hippocampal plasticity and memory [134], the mechanistic understanding of myokine-related effects on the brain is still insufficient [135]. In the following sections some of the myokines—and other factors released from muscle upon exercise—with known neuroprotective effects are briefly discussed and their effects on brain mitochondria outlined (see Figure 2).

#### 5.2.1. Irisin

Irisin is an exercise-induced myokine released by the cleavage of the membrane-bound precursor protein fibronectin type III domain-containing protein 5 (FNDC5), a transmembrane precursor protein expressed in muscle under the control of PGC-1α [136]. Aerobic exercise is a potent stimulus for its secretion [137,138]. Irisin exerts its beneficial effects mainly via acting on mitochondria. These effects, however, are better understood in adipose tissue, the heart, the lung, and the liver than in the brain. Irisin has been first identified as a factor involved in adipocyte browning by stimulating mitochondrial uncoupling protein 1 [139]. In the lung, irisin protects mitochondria from ischemia-reperfusion injury by interacting with mitochondrial uncoupling protein 2, resulting in reduced oxidative stress [140]. It was later shown to be cardioprotective by increasing the activity of the antioxidative enzyme superoxide dismutase (SOD) and restoring mitochondrial localization to SOD2 [141]. Recently, it was demonstrated that irisin prevents excessive oxidative stress and aberrant mitochondrial fission, while promoting mitochondrial biogenesis in hepatocytes after ischemia/reperfusion [142].

The increased understanding of the actions of irisin on the brain was recently summarized [143]. Briefly, FNDC5/irisin is also expressed in the hippocampus, a brain region involved in learning and memory, where irisin stimulates the expression of BDNF [144] (see below). The effect of irisin released from muscle on the brain is not entirely clear yet, but it includes disinhibition of BDNF (through histone deacetylase (HDAC)-mediated inhibition) in the brain [145]. Supporting this notion, regular swimming exercise (1 h per day, 5 days per week for 5 weeks) has been shown to be protective in a mouse model of AD, with the FNDC5/irisin-mediating exercise effects of reducing neurodegeneration, enhancing synaptic plasticity, and ameliorating memory deficits [136].

#### 5.2.2. Cathepsin B

Cathepsin B (CTSB) is a lysosomal cysteine protease secreted by muscle in response to exercise with beneficial effects on cognition [135]. Its release has been shown to be linked to PGC-1α expression [146]. Similarly to those of irisin, the positive effects of CTSB seem to be partially mediated by increased BDNF expression [147].

CTSB is involved in mitochondrial cell death signalling by regulating the release of proapoptotic molecules [148,149], at least in certain cell types. Accordingly, the inhibition of CTSB has been shown to be beneficial in models of AD and other brain disorders, as recently reviewed [150]. In mice, CTSB has been shown to be necessary for running-induced adult neurogenesis and memory improvements [135].

#### 5.2.3. BDNF

BDNF signalling plays a major role in neurogenesis and in the process of learning and memory formation [147,151]. Exercise enhances BDNF release in the brain [152], supporting exercise-induced learning and memory benefits [153]. BDNF is also expressed by skeletal muscle satellite cells and plays a key role in maintaining muscle progenitors cells [154] but also muscle nerve development and maintenance [155]. BDNF is also released from muscle, but this effect may be sex specific, as at least in response to fasting, it was observed in female but not male mice [156]. In exercising humans, this sex difference has not been described, with serum BDNF levels increasing following exercise in an intensity-dependent manner [157]. This may affect the brain as BDNF readily crosses the BBB.

BDNF has been reported to exert effects on mitochondria. It activates AMPK, enhances fatty acid oxidation [156,158], and prevents a decline of mitochondrial mass in response to fasting [156] in muscle cells. It is noteworthy that BDNF has further been demonstrated to increase mitochondrial biogenesis in cultured murine hippocampal neurons [159]. Decreased levels of BDNF have been reported in AD patients’ brains [160], as recently reviewed [161]. Accordingly, increased BDNF levels (together with enhanced adult neurogenesis) have been shown to constitute an important part of the beneficial effects of exercise on cognition in AD [162].

#### 5.2.4. FGF21

Several myokines are induced by mitochondrial stress or modulate mitochondrial function. They are referred to as mitokines if released in response to perceived mitochondrial stress and in turn produce beneficial effects in distal tissues [163].

Among muscle-derived mitokines is FGF21, the serum level of which is increased by acute exercise [164], and which is implicated in the regulation of mitophagy [165], mitochondrial dynamics [166], and possibly mitochondrial biogenesis [167]. Similar to irisin and CSTB, FGF21 expression is regulated by PGC-1α [168]. Importantly, FGF21 can penetrate the BBB [169], but whether its effects on the brain are direct or indirect is not well understood [170]. FGF21, however, has been reported to exert protective effects on the BBB [171], and FGF21 signalling has been demonstrated to regulate behavioural and metabolic adaptations to food alteration and restriction [172]. The beneficial effects of FGF21 have also been observed in cellular and murine models of AD [173] and PD [174].

#### 5.2.5. Humanin

Another group of myokines comprises molecules released from mitochondria in response to exercise that may then exert systemic effects, such as the mitochondrially encoded, apoptosis-suppressing [175] peptide humanin [176,177]. It is noteworthy that humanin has been shown to be neuroprotective in cellular models of AD [178,179] and PD [180,181], likely via preventing mitochondrial dysfunction and the initiation of aberrant mitochondrial cell death signalling.

#### 5.2.6. Cytokines

Regular exercise and high cardiorespiratory fitness are associated with reduced markers of general inflammation [182]. Mechanistically, this effect may be induced by the exercise-induced release of cytokines [183,184]. Among these cytokines is interleukin-10 (IL-10), a potent anti-inflammatory signalling molecule that in the brain, for example, modulates astroglial activation and neuroinflammation [185]. In macrophages, IL-10 has been shown to directly regulate mitochondrial dynamics and respiration via mitochondrial arginase-2 [186]. Neuroprotective IL-10 effects are therefore likely partially mediated by the metabolic reprogramming of mitochondria.

Another exercise-induced cytokine (and classical myokine [187]) with prominent effects on the brain, and brain mitochondria, is interleukin-6 (IL-6) [188]. While IL-6 has been shown to regulate mitochondrial biogenesis in astrocytes [189], its mode of action is complex and can result in both pro- and anti-inflammatory outcomes [183,190].

Interestingly, polymorphisms in both IL-6 and IL-10 have been linked to an increased risk to develop AD [191,192]. The genetic transfer of human IL-10 has been shown to be neuroprotective in a rat model of PD by reducing neuroinflammation [193], while IL-6 deficiency was associated with increased neurodegeneration in a mouse model of PD [194].

Beneficial exercise effects are likely mediated in part via adaptations to proinflammatory signals. The quality and intensity of the exercise stimulus determine inflammatory responses resulting from muscle damage, with, for example, eccentric exercise resulting in particularly high systemic inflammation [195]. An initial proinflammatory phase after muscle damage is followed by adaptive processes that reduce inflammation [121]. Whether high intensive exercise and associated pronounced mitochondrial damage in skeletal muscle [78] and resulting inflammation affect the brain, as suggested by investigations on ultramarathon runners [196], and which consequences this might have on the pathogenesis of neurodegenerative diseases, remains to be elucidated. Experimental evidence from studies in rats, however, indicates the adverse consequences of overtraining on cognitive function [197].

### 5.3. Metabolites

While exercise exerts largely positive effects on brain metabolism, aging is associated with metabolic deficits that may be involved in the development of neurodegeneration [198]. Several metabolites are involved in cellular exercise responses, and some muscle-derived metabolites may physically reach the brain or exert indirect effects on the brain. Here, we focus on lactate, but other metabolites are certainly involved in muscle–brain communication.

Lactate levels in the brain are regulated mainly by astrocytes that provide lactate as a substrate for energy metabolism to neurons [21]. Transport of lactate across the brain endothelium conversely depends strongly on endothelial cells [199]. In response to intensive exercise, lactate is released from the skeletal muscle and leads to increased lactate levels in blood [200]. This impacts brain metabolism and correlates with lactate uptake and oxidation in the brain parenchyma [201] as well as with increased the excitability of the primary motor cortex [202]. Exercise-induced increased systemic lactate levels thus may be involved in the brain effects of exercise, as has been demonstrated for cerebral angiogenesis [200] and has been hypothesized for exercise-triggered neurogenesis [203,204]. The molecular mechanism of the lactate-mediated beneficial effects of exercise on the brain may again depend at least partially on BDNF signalling [205], which is in line with the potential effects on neurogenesis and is relevant for neurodegenerative diseases; adult neurogenesis is impaired, for example, in AD and PD [206]. Lactate also induces the upregulation of neuroangiogenesis through brain vascular endothelial growth factor (VEGF) [200] and the synaptic plasticity genes cfos, Arc, and Zif [207].

Additionally, the citric acid cycle metabolite succinate has recently been shown to be released into the skeletal muscle interstitium and into the systemic circulation in response to exercise to exert paracrine effects [208]. Whether this succinate reaches the brain and how the brain might profit from this are open questions. The reported anti-inflammatory effects of extracellular succinate in the brain [209] indicate that neurons could potentially benefit from the higher availability of this metabolite.

### 5.4. MicroRNAs

MicroRNAs are short noncoding RNAs that can repress gene expression post-transcriptionally by binding complementary RNAs. The regulation and functions of microRNAs have been reviewed in detail elsewhere [210].

Muscle-tissue-specific microRNAs have been referred to as “myomiRs” and are thought to be key elements in skeletal muscle adaptations in response to contraction [211]. Alterations of circulating microRNAs (c-miRNAs) in response to exercise, depending on the quality and extent of the exercise stimulus, suggest that microRNAs are involved in systemic adaptations to exercise as well [212,213]. microRNAs play a role in angiogenesis [214], inflammation [215], the regulation of muscle contraction [216], the response to hypoxia [217], and mitochondrial metabolism [218]. The interplay of microRNAs with mitochondria and redox regulation is also important in the brain [219].

The patterns of c-miRNAs regulation differ. Some c-miRNAs are upregulated in response to acute exercise and after (miR-146a, miR-222), some to acute exercise but not after (miR-21 and miR221), some are responsive only to sustained training (miR20), and some are nonresponsive (miR-133a, miR-210 and miR-328) [220].

Future systematic investigations will be necessary to determine the mechanistic role of such microRNAs [213,219], particularly in the communication with tissues distal from muscle, such as the brain.

### 5.5. Direct Neuroprotective Signaling from Skeletal Muscle Mitochondria?

It is becoming increasingly clear that mitochondria not only control myriad intracellular processes but that they influence mitochondria and cells in distant localizations and thus have important communication roles also across cellular boundaries. They even appear to be synchronized across cells without contact [221], a phenomenon that is not yet well understood.

Mitochondria not only release and react to signalling molecules, but they can also be transferred between cells as whole organelles or organelle components [222,223]. This “mitochondrial transfer” has also been shown to have functional benefits, as it can rescue respiration in recipient cells [224,225,226,227]. Such transfer of mitochondria between different cell types includes, for example, the exchange of mitochondria between astrocytes and neurons to assist with the degradation of damaged neuronal mitochondria [228] or to support neurons with astrocytic mitochondria, e.g., after a stroke [229,230]. Furthermore the delivery of exogenous isolated mitochondria [231] or mitochondrial transfer from exogenous stem cells [232] has resulted in beneficial outcomes for brain tissues.

It is still debated how mitochondria are transferred between cells, but several routes have been suggested. Apart from the direct release and uptake of mitochondria or their components, transfer via tunnelling nanotubes to adjacent cells has been suggested as well as transfer in extracellular vesicles, which is associated with a reduced release of inflammatory factors as compared to the transfer of “naked” mitochondria [233]. Importantly, the packaging of mitochondrial proteins in extracellular vesicles has recently been demonstrated to be selective, with damaged proteins being transported directly to lysosomes [233]. It is possible that mitochondrial components may also be transported by extracellular vesicles from skeletal muscle to the brain, as the release of extracellular vesicles from skeletal muscle into the systemic circulation occurs [120] particularly in response to exercise [118]. Currently, however, the assessment of the origin of extracellular vesicles is challenging [118], making the role of mitochondrial transfer in exercise-induced brain effects speculative. Interestingly, the systemic administration of isolated mitochondria into the blood resulted in the relocalization of the exogenously administered mitochondria in the brain and has been shown to be beneficial in rodent models of AD [234] and PD [235]. We are not aware of studies showing the involvement of mitochondrial transfer from skeletal muscle to the brain in exercise-dependent benefits on neurodegenerative diseases, but this is a possibility that merits being investigated.

Taken together, although the mechanisms of muscle–brain signalling are incompletely understood, regular exercise exerts clear beneficial effects on the brain. These include molecular alterations, such as reduced ROS production and oxidative damage, as well as improved enzymatic antioxidant defences [104] and prominent effects on components involved in mitochondrial biogenesis [103,104] and on hippocampal adult neurogenesis [236]. The role of mitochondria and mitochondrial stress in skeletal muscle in response to exercise on brain function is yet to be clearly delineated. While mitochondria are importantly involved in the response of skeletal muscle to exercise, it remains to be elucidated how they participate in systemic signalling, particularly to the brain. For example, the roles of mitokines and of mitochondrial transfer in exercise signalling in general, and specifically to the brain, are exciting topics for future research.

**Figure 3:**
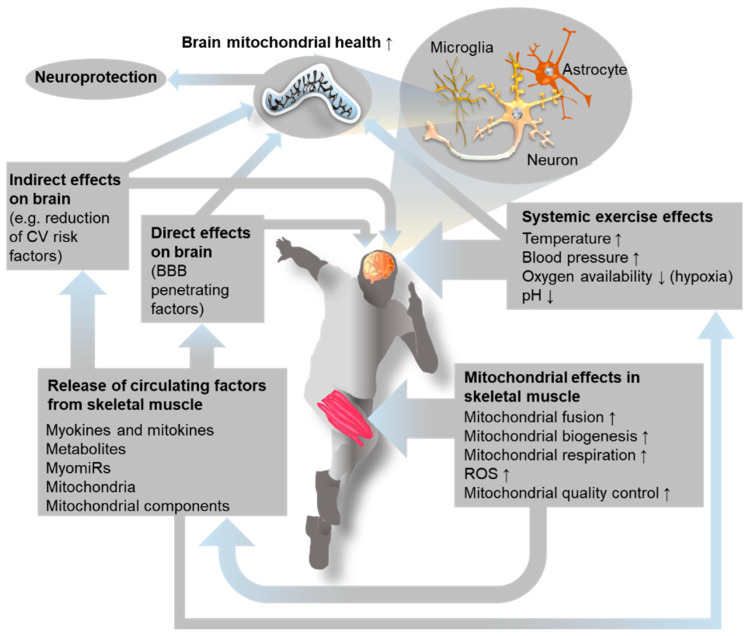
Factors controlling exercise-induced communication from muscle to brain: the central role of mitochondria. CV: cardiovascular; BBB: blood–brain barrier; ROS: reactive oxygen species; MyomiRs: microRNAs released from muscle.

In summary, the benefits conferred onto the brain by exercise of the skeletal muscles may be protective for numerous neurological pathologies, including notably AD, PD, and other neurodegenerative diseases [127]. A summary of the potential routes of muscle–brain communication is provided in Figure 3.

## 6. Strategies to Boost Brain Mitochondria by Exercise

Although many open questions remain on the mechanistic details of the exercise-induced benefits on brain mitochondria, the potential of exercise to prevent neurodegeneration is immense. Regular exercise improves brain function, including the cognitive function [107] and hormonal activity [106] of the brain. A direct outcome of exercise—improved cardiorespiratory fitness—has been shown to protect from neurodegeneration risk factors by enhancing, for example, resilience against disruptions of CBF [237]. High cardiorespiratory fitness is also associated with reduced cognitive dysfunction in older individuals [238]. A challenge for the clinical application of exercise as a medicine is the selection of the appropriate exercise regimen, depending both on the capacities of the patients or at-risk populations and the aim. Training plans to this end should be designed with the assistance of sports science specialists [239] in order to both maximize health-promoting effects (selection of adequate exercise parameters) and exclude any risks arising, e.g., from pre-existing morbidities or exercise loads that are too high for an individual.

The WHO guidelines “Global Recommendations on Physical Activity for Health” and the recommendations from the American College of Sport Medicine (ACSM) both propose at least 150 min per week of moderate intensity (endurance) or 75 min of vigorous physical exercise (e.g., intense interval exercise) or an equivalent combination for health benefits in older adults [240,241]. Strength and balance exercise on three or more days per week is recommended for people to maintain force and to prevent falls.

Different exercise modalities and “doses” exert distinct benefits [242], including on the mitochondrial level [243]. The main outcome of an exercise modality is determined by the type, intensity, and frequency/duration of the exercise. Endurance training (main effects on oxidative capacity and fatigue resistance), for example, has a particular potential to improve mitochondrial biogenesis and cardiorespiratory fitness.

For trained [244] and untrained [245] healthy individuals, as well as for deconditioned lung cancer patients [246], time-efficient interval trainings may be equally beneficial or superior to moderate-intensity continuous training (MICT). Interval training programs are commonly classified as high-intensity interval training (HIIT; with intensities close to maximum capacities) and sprint interval training (SIT; ‘supramaximal’ intensities), with MICT, HIIT, and SIT all improving aerobic capacity and mitochondrial content in skeletal muscle. SIT can induce mitochondrial biogenesis and improve aerobic capacity similar to MICT despite smaller durations, while HIIT can improve aerobic capacity more efficiently than MICT [247]. Importantly, for the selection of optimal exercise parameters, the baseline individual characteristics must be considered, with high intensity alternatives potentially not being suited for certain aged populations and patient groups.

Resistance training (increasing strength and muscle fibre cross-sectional area) can be applied to boost the function of selected muscles [247]. Intriguingly, training modalities also affect mitochondrial functions differentially [70,248,249], although this phenomenon is insufficiently understood, especially in tissues other than skeletal muscle. Enhanced mitochondrial biogenesis is one of the earliest observed [68] and most studied effects of endurance exercise. The effects of resistance exercise on muscle mitochondria have been less frequently investigated but may be associated with smaller biogenesis effects and instead with greater improvements of mitochondrial respiration [250]. The not yet fully understood effects of resistance exercise that may be beneficial in neurodegenerative diseases have been summarized recently [251]. The combination of resistance with endurance exercise has been shown to be particularly effective to boost mitochondrial respiration, at least in muscle [99].

Taken together, performing different types of exercises exerts specific effects on skeletal muscle mitochondria and affects performance differentially. Although this is an important characteristic to harness physical exercise as a treatment for neurological diseases, the effects of specific exercise parameters on brain and brain mitochondria are still poorly understood. Increasing the complexity of the field, the contributions of mitochondria in different brain cell types (neurons, glial cells, endothelial cells, etc.) to the beneficial effects of different exercise modalities remain to be better explored.

The potential reduction of aerobic exercise effects due to combination with strength training [252] further highlights the importance of well-designed exercise programs with adequate training loads and structures and sufficient regeneration times. The selection of individually effective training programs is key for exercise benefits and is also important to consider for clinical studies.

While it has been shown for former athletes that team and contact sports yield benefits on overall mortality similar to endurance exercise [253], risk of traumatic brain injury from, for example, contact sports [253] or—more debated [254] —team sports, such as football [255], increases the risk for neurodegenerative diseases later in life. In contrast, boxing—performed as a noncontact sport—for example, is explored as an intervention, e.g., for PD [244,245,256].

## 7. Conclusions

Regular exercise benefits mitochondria in skeletal muscles but also in remote tissues, such as the brain. Compromised mitochondria are probably key players in the pathogenesis of numerous neurodegenerative diseases including PD and AD. Exercise, in theory, may thus be an excellent preventive strategy. This assumption is confirmed by numerous studies, as recently reviewed for PD [6] and AD [5]. While regular endurance exercise seems to be the most efficient strategy to improve mitochondrial biogenesis and aerobic capacity, its combination with resistance exercise may have a synergetic effect to maximize energy metabolism outcomes. The release of numerous signalling molecules, including myokines, from exercising muscles is associated with prominent neuroprotective effects. The release of these factors depends on the exercise modality, intensity, frequency, and duration. A well-designed mix of regular exercise of different modalities is thus expected to be the best for enhancing different mitochondrial functions in the brain. The combination of different exercise modalities (combining endurance and strength training, and including, for example, exercise to improve balance) may also be most useful to slow down the loss of muscle mass and physical capacities that are major factors for decreased quality of life in patients with neurodegenerative diseases [257].

Importantly, acute intensive exercise also involves risks such as injury, oxidative stress, and acutely reduced immune functions [182]. These risks increase at an older age and must be taken into account, especially when beginning a new training routine. Conversely, regular moderate exercise has the potential to markedly improve both antioxidative and immune capacities [182].

Despite the well-known benefits of regular exercise on brain mitochondria, the routes of communication from mitochondrial adaptations in the contracting skeletal muscle to the brain are still poorly understood. Here, we summarized several potential components of the muscle–brain axis, including various signalling molecules and potential direct mitochondrial transfer. Future investigations are needed to determine the contributions and potentially differential roles of the outlined routes from muscle to the brain. This will help to establish efficient pharmacological means to modulate the muscle–brain axis in order to strengthen brain mitochondria as a preventive measure against neurodegenerative diseases.

## Data Availability

No new data were created or analyzed in this study. Data sharing is not applicable to this article.
